# Validity and Reliability of a French Version of the Empty Nose Syndrome 6-Item Questionnaire

**DOI:** 10.3390/jcm15020791

**Published:** 2026-01-19

**Authors:** Antonino Maniaci, Jérôme R. Lechien

**Affiliations:** 1 Research Committee of Young Otolaryngologists, International Federation of Otorhinolaryngological Societies (IFOS), 75009 Paris, France; antonino.maniaci@unikore.it; 2 Faculty of Medicine and Surgery, University of Enna Kore, 94100 Enna, Italy; 3 Department of Surgery, UMONS Research Institute for Health Sciences and Technology, University of Mons (UMons), B7000 Mons, Belgium; 4 Division of Laryngology and Broncho-Esophagology, Department of Otolaryngology-Head Neck Surgery, EpiCURA Hospital, UMONS Research Institute for Health Sciences and Technology, University of Mons (UMons), B7000 Mons, Belgium; 5 Department of Otorhinolaryngology and Head and Neck Surgery, Foch Hospital, Paris Saclay University, Worth Street, 40, 92150 Suresnes, France; 6 Department of Otorhinolaryngology and Head and Neck Surgery, Elsan Hospital, 70000 Poitiers, France

**Keywords:** empty nose syndrome, ENS6Q, rhinology, nose, evaluation, management, otolaryngology, otorhinolaryngology, French, head neck, surgery

## Abstract

**Objective**: To validate a French version of the Empty Nose Syndrome 6-Item Questionnaire (Fr-ENS6Q). **Methods**: Thirty-three patients with a diagnosis of empty nose syndrome (ENS) and 50 healthy individuals completed the Fr-ENS6Q and the French versions of the Sinonasal Outcome Tool-22 (Fr-SNOT22) and Nasal Obstruction Symptom Evaluation (Fr-NOSE). The internal consistency (Cronbach-α), test–retest reliability (intraclass correlation coefficient (ICC)), and external validity (correlations between Fr-ENS6Q, Fr-SNOT-22, and Fr-NOSE) were evaluated. The threshold of Fr-ENS6Q for suspecting ENS diagnosis was calculated using the receiver operating characteristic (ROC) curve. Depression and anxiety were investigated with the General Anxiety Disorder-7 (GAD-7) and the Patient Health Questionnaire-9 (PHQ-9). **Results**: The Cronbach-α of Fr-ENS6Q was 0.81, indicating an adequate internal consistency. Patients reported higher ENS6Q scores than controls, indicating high internal validity. The Fr-ENS6Q was significantly correlated with the Fr-NOSE (r_s_ = 0.56; *p* = 0.001) and Fr-SNOT22 (r_s_ = 0.67; *p* = 0.001), which supports a high external validity. The test–retest reliability was high for ENS6Q scores (ICC = 0.895; 95%CI: 0.763–0.971). An ENS diagnosis can be suspected with an Fr-ENS6Q cutoff ≥ 12 for French-speaking ENS patients. Depression was detected in 97% of patients, with 84.9% requiring further assessment. Patients reported missing symptoms in the ENS6Q, such as sleep disturbance related to nasal airflow disorder, face/eye/dental pain, and fresh nasal sensations during airflow. **Conclusions**: The Fr-ENS6Q is a valid and reliable patient-reported outcome questionnaire for assessing the severity of ENS symptoms in the French-speaking population. Further improvements to the ENS6Q could consider the inclusion of symptoms that are not present in the current ENS6Q.

## 1. Introduction

The term “empty nose syndrome” (ENS) was originally proposed by Kern and Stenkvist in 1994 to describe the empty space in the region of the inferior and middle turbinates on coronal computed tomography images of patients who had undergone inferior and middle turbinectomies [1]. The pathophysiology of ENS remains unelucidated, with the identification of several abnormalities in the nasal airflow dynamics, air humidification, warming, mucociliary clearance, and trigeminal-related sensory function in some patients [2,3]. However, it remains unclear why some patients with anatomical turbinate defects develop ENS, while others do not experience symptoms with similar anatomy [3]. The primary symptoms of ENS include paradoxical nasal obstruction, dyspnea, suffocation, burning, crusting, and dryness, and they commonly impact the patient’s quality of life (QoL) [4]. In 2017, Velasquez et al. developed the Empty Nose Syndrome 6-Item Questionnaire (ENS6Q), which is a validated and standardized patient-reported outcome questionnaire evaluating the severity of ENS symptoms [4]. ENS6Q is validated in English. To date, there is no validated version for French-speaking countries, which include more than 400 million inhabitants.

This study aimed to validate a French version of the ENS6Q (Fr-ENS6Q).

## 2. Materials and Methods

### 2.1. Ethical Statement

The study protocol was approved by the CHU Saint Pierre Ethics Board (approval code: CE220217). Informed consent was obtained from patients and healthy individuals. This study adhered to the STROBE guidelines for observational studies to ensure transparency and replicability of our findings [5].

### 2.2. Empty Nose Syndrome 6-Item Questionnaire

**Figure 1:**
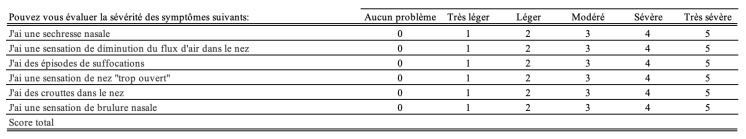
The French version of the Empty Nose Syndrome 6-Item Questionnaire. The patient can complete the following questionnaire. The score ranges from 0 to 30.

Two board-certified otolaryngologists and a linguist developed the Fr-ENS6Q from the English version of the ENS6Q [4]. The Fr-ENS6Q was developed following established cross-cultural adaptation guidelines. Two independent forward translations were performed by board-certified otolaryngologists and reconciled by consensus with a linguist to ensure conceptual equivalence. The preliminary version underwent cognitive pretesting with 5 ENS patients who evaluated readability and comprehension. Following patient feedback integration, two independent native English speakers performed backward translation. The expert committee (otolaryngologists, linguists, and patients) reviewed all versions to confirm semantic, idiomatic, and conceptual equivalence with the original ENS6Q (Figure 1).

### 2.3. Patients and Setting

The Fr-ENS6Q was proposed to French-native ENS patients and healthy individuals (control group). Given the rarity of the disorder, the patients were recruited with the help of the patient organization “*victimes du SNV*” between March 2024 and July 2024. ENS patients had a clear diagnosis of ENS, which was based on a history of nasal surgery, tomodensitometry, and, for some of them, the cotton test [4]. Patients without a clinical or imaging diagnosis of ENS were excluded. The control group included individuals without ear, nose, and throat symptoms or findings. Subjects with chronic alcohol or tobacco consumption, psychiatric disorders, or an inability to understand the aim of the study were excluded.

### 2.4. Symptom and Nasal Evaluations

The patients and healthy individuals completed the Fr-ENS6Q, the French versions of the Sinonasal Outcome Tool-22 (Fr-SNOT-22) [6], and the Nasal Obstruction Symptom Evaluation (Fr-NOSE) [7]. Experts also evaluated the anxiety and depression symptoms of patients with the French versions of the General Anxiety Disorder-7 (GAD-7) [8] and the Patient Health Questionnaire-9 (PHQ-9) [9]; both were assessed at baseline during an out-of-crisis period. GAD-7 is a validated and standardized patient-reported outcome questionnaire evaluating the severity of anxiety in patients from 0 to 21. The minimal, mild, moderate, and severe anxiety scores were 0–4, 5–9, 10–14, and 15–21, respectively [8]. PHQ-9 is a patient-reported outcome questionnaire adapted from the Diagnostic and Statistical Manual of Mental Disorders, 4th edition (DSM-IV). PHQ-9 measures the severity of depression with minimal, mild, moderate, moderately severe, and severe depression scores of 1–4, 5–9, 10–14, 15–19, and 20–27, respectively [9].

#### Statistical Analyses

The statistical analyses were performed with Statistical Package for the Social Sciences for Windows (SPSS version 30.0; IBM Corp., Armonk, NY, USA). Subjects completed the Fr-ENS6Q twice over 14 days (range: 13.0–15.0 days; mean time interval: 14.0) to assess the test–retest reliability with the intraclass correlation coefficient (ICC). The Cronbach-α was used to evaluate the internal consistency. The external validity was evaluated through a Pearson correlation analysis between Fr-ENS6Q, Fr-SNOT-22, and Fr-NOSE. The internal validity was evaluated by comparing the Fr-ENS6Q scores between ENS patients and asymptomatic individuals (Mann–Whitney *U* test).

The association between Fr-ENS6Q, GAD-7, and PHQ-9 was studied with the Spearman correlation coefficient regarding the data distribution. The threshold of ENS6Q was determined with the receiver operating characteristic (ROC) curve. A level of significance of *p* < 0.05 was used.

## 3. Results

Thirty-three ENS patients completed the evaluations. There were 14 females (42.4%) and 19 males (57.6%). The mean age was 41.9 ± 12.7 years. The diagnosis approaches are reported in Table 1. The diagnosis was based on nasofibroscopy in 16 cases (48.5%). In 17 cases (51.5%), a tomodensitometry was carried out to exclude other comorbidities and to support the diagnosis. The cotton test was performed on 16 patients (48.5%; Table 1). Thirty-two patients (97%) reported an ENS6Q score ≥ 11, which supported the diagnosis according to the threshold defined by Velasquez et al. [4]. The patient with an ENS6Q inferior to the cutoff had a confirmed diagnosis despite an ENS6Q at 8/30. Note that seven patients (21.2%) made the diagnosis themselves before visiting a physician who confirmed it. Most patients developed ENS after a septoplasty with bilateral inferior turbinoplasties (36.4%), which was associated with a functional endoscopic sinus surgery for chronic rhinosinusitis in 27.3% of cases (Table 1). The primary comorbidities of patients included suspected laryngopharyngeal reflux disease (27.3%), irritable bowel syndrome (24.2%), allergy (24.2%), asthma (21.2%), and migraine (21.2%). Twenty-one patients (63.6%) reported that they developed severe sleep disorders after the nasal surgery associated with the development of ENS. The control group included 12 males and 38 females. The mean age was 46.6 ± 15.9 years. Fourteen individuals had an allergy (28%). The most prevalent comorbidities in the control group included gastroesophageal reflux disease (N = 9; 18%), hypertension (N = 8; 16%), hypercholesterolemia (N = 8; 16%), and asthma (N = 6; 12%).

The Cronbach-α of Fr-ENS6Q was 0.81 (95%CI: 0.74, 0.87), indicating an adequate internal consistency. The prevalence of symptoms and the mean symptom scores of Fr-ENS6Q, Fr-NOSE, and Fr-SNOT-22 in ENS and control populations are reported in Table 2.

The item and total scores of Fr-ENS6Q patients were significantly higher compared to controls, indicating a high internal consistency. The patients reported that they had the following symptoms that are not included in the ENS6Q: nasal cold/fresh sensations during airflow (N = 27, 81.8%); face, eye, ear, or dental pain (N = 29; 87.9%); sensation of nasal blockage or obstruction (N = 27; 81.8%); blocked ears (N = 27; 81.8%); sleep disturbances related to nasal airflow disorder (N = 32; 97.0%); hyperventilation (N = 26; 78.8%); and sport limitation related to nasal airflow disorder (N = 30; 90.9%).

The psychological distress investigation (PHQ-9) reported a depression rate of 97%, with 84.9% of patients requiring assessment according to the PHQ-9 criteria (Table 3) [9]. An abnormal anxiety was found in 87.9% of patients, with 72.7% requiring medical assessment regarding GAD-7 criteria [8]. The Fr-ENS6Q was significantly correlated with the Fr-NOSE (r_s_ = 0.56; *p* = 0.001) and the Fr-SNOT22 (r_s_ = 0.67; *p* = 0.001), which supports a high external validity. The Fr-ENS6Q reported high correlation coefficients with PHQ-9 (r_s_ = 0.46; *p* = 0.001) and GAD-7 (r_s_ = 0.57; *p* = 0.001; Table 4). Suffocation and the sensation of a nose being too open were the symptoms most associated with GAD-7 and PHQ-9 scores (Table 4).

**Figure 2:**
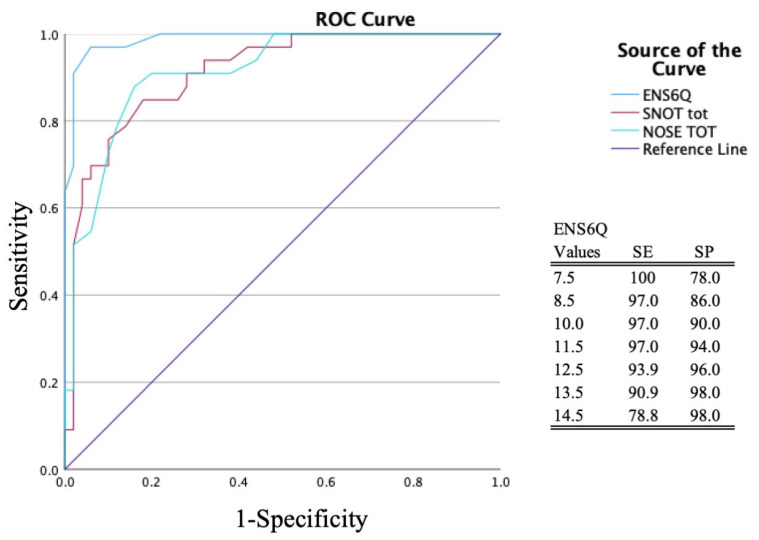
Receiver operating characteristic curve. The Fr-ENS6Q curve was significantly better than the Fr-SNOT-22 and Fr-NOSE curves. The threshold associated with the highest sensitivity and specificity was 11.5 (≥12).

The test–retest reliability was high for ENS6Q scores (ICC = 0.895; 95%CI: 0.763–0.971). The threshold of Fr-ENS6Q for suspecting ENS diagnosis was calculated using the receiver operating characteristic (ROC) curve (Figure 2). The area under the curve (AUC) was 0.989 (95% CI: 0.969–1.000) for Fr-ENS6Q, 0.841 (95% CI: 0.753–0.929) for Fr-SNOT-22, and 0.825 (95% CI: 0.733–0.917) for Fr-NOSE, confirming Fr-ENS6Q’s superior diagnostic discrimination (*p* < 0.001).

The Fr-ENS6Q was more discriminating than Fr-SNOT-22 and Fr-NOSE for determining ENS diagnosis. An ENS diagnosis can be suspected with an Fr-ENS6Q cutoff ≥ 12 for French-speaking ENS patients. This threshold was associated with a sensitivity of 97.0% (95% CI: 84.2–99.9%) and specificity of 94.0% (95% CI: 83.5–98.7%).

## 4. Discussion

The development of a valid and reliable French patient-reported outcome questionnaire is important for improving the management of French patients with ENS.

The findings of the present study show that the Fr-ENS6Q is a valid and reliable clinical instrument for measuring the severity of ENS symptoms and for discriminating ENS patients from healthy individuals. The Cronbach-α of Fr-ENS6Q was 0.81, which can be statistically considered an adequate internal consistency value [10]. The Fr-ENS6Q Cronbach-α found in the present study corroborates the values found in the validity studies of other clinical instruments, such as the English version of ENS6Q (0.93) [4], the Fr-SNOT-22 (0.93) [6], the Fr-NOSE (0.86) [7], or the rhinosinusitis quality of life survey (0.57–0.83) [7].

The present study investigated the external validity through the relationship between ENS6Q and SNOT-22. Our data suggest that both questionnaires are significantly correlated (r_s_ = 0.67). The external validity of the English version of ENS6Q was not evaluated by Velasquez et al. [4], which limits our comparison with the literature. However, considering other common nasal patient-reported outcome questionnaires, the external validities of Fr-NOSE (r_s_ = 0.40) [7] and Fr-SNOT-22 (r_s_ = 0.64) [6] were lower than the value found for the Fr-ENS6Q.

Velasquez et al. observed significant differences between ENS patients, asymptomatic individuals, and patients with chronic rhinosinusitis with nasal polyps for the SNOT-22 and ENS6Q scores, which indicates adequate internal validity [4]. In this study, we limited the study populations to ENS patients and healthy individuals. The ENS6Q comparison between both populations showed that all ENS6Q items and the total score were significantly higher in ENS patients compared to controls. Finally, the test–retest reliability of the Fr-ENS6Q was high (ICC = 0.895), which was similar to the English version of the ENS6Q (ICC = 0.96) [4].

The psychological evaluations of the present study suggest that 84.9% of patients had severe or moderately severe depression, requiring psychological evaluations by practitioners [9]. In the same way, 72.7% of patients had moderate or severe anxiety on the GAD-7 scores, meaning that they were at risk of long-term physical health problems [8]. The Fr-ENS6Q was significantly associated with the GAD-7 and PHQ-9 scores, which confirms the severe impact of ENS symptoms on the patient’s quality of life, especially due to suffocation and the sensation of a nose being too open. Although some external confounding factors may have occurred in our cohort (familial or workplace environment stress/psychological burden), our observation of the significant impact of ENS symptoms on patients’ quality of life and mental health corroborates those of previous studies [11,12,13]. In the study of Manji et al., 67.9% of ENS patients had moderately severe or severe depression on the PHQ-9, with 67.9% requiring psychological assessment [12]. In 2021, Kim et al. [11] investigated the depression prevalence and stress level in 24 ENS patients. Using the Beck Depression Inventory thresholds, the authors reported that 71% of patients had several degrees of depression [11]. The high rate of mental health distress in ENS patients was similarly shown by Lamb et al., who reported that 53% of ENS patients met diagnostic thresholds for somatic symptom disorder on the PHQ [13]. The consideration of depression and anxiety is important in the clinical evaluation of ENS patients, as suicidal thoughts have been reported in 37.1% of cases [14]. Importantly, Huang et al. observed that partial or total relief of ENS symptoms after surgical procedures can reduce depression symptoms by more than 70% [15].

In this study, 63.6% of patients complained of severe sleep disturbance since the onset of the ENS, while 97% had mild-to-severe sleep disturbance. The SNOT-22 scores of ENS patients were particularly high. The medical history also showed that patients commonly have symptoms that are not included in the ENS6Q, e.g., nasal cold/fresh sensations; face, eye, ear, or dental pain; sensation of nasal blockage or obstruction; blocked ears; hyperventilation; and exercise limitation related to nasal airflow disorders. The lack of consideration of these symptoms is the primary limitation of ENS6Q because these symptoms concern a substantial number of patients, contributing to the development of psychological distress. This observation was supported by Huang et al., who found that depression was driven in ENS patients by ear and facial symptoms, psychological dysfunction, and sleep dysfunction according to the SNOT-25 outcomes [16]. Considering these symptoms in a revised ENS6Q or another ENS patient-reported outcome questionnaire makes sense to improve clinical evaluation at baseline and throughout treatment.

The primary limitation of the present study is the small number of ENS patients. However, ENS is a rare condition, which makes it difficult to recruit a large cohort population. For this reason, we collaborated with a French-speaking patient organization of patients with a confirmed ENS diagnosis.

To the best of our knowledge, there is no French version of ENS6Q evaluating the severity of ENS symptoms. The validation of a French version of ENS6Q is therefore the primary strength of this study. The ROC analysis suggested a cutoff of ≥12 for French-speaking ENS patients. The evaluation of other symptoms associated with ENS is another strength of this study because it can lead to the development of a more complete questionnaire of ENS symptoms. The primary limitation is the moderate-to-high value of test–retest reliability (0.81), which is lower than the English version. However, according to recommendations [17], this value is still consistent for the patient-reported outcome questionnaire. The small number of ENS patients, which is related to the rarity of the disease, is an additional limitation, as it is commonly recommended to have ≥5–10 subjects per item to validate a patient-reported outcome questionnaire. Another challenging point highlighted in the literature is the diagnosis of ENS. Future diagnoses of empty nose syndrome will likely integrate objective measures with existing subjective assessments to address current diagnostic limitations. Emerging objective approaches include computational fluid dynamics modeling for airflow analysis and identifying resistance patterns and symmetry alterations, as well as specific CT scan features such as compensatory mucosal hypertrophy [18,19]. A standardized diagnostic framework may combine these tools with refined physical examination, validated questionnaires including the ENS6Q, and comprehensive clinical and psychological evaluation, reflecting the multifactorial complexity of this syndrome. A critical limitation of this validation study is the absence of a disease control group, including patients with chronic rhinosinusitis or chronic rhinitis. While we intentionally excluded such controls to avoid confounding from overlapping conditions like laryngopharyngeal reflux disease (which can cause nasal crusting, dryness, and inflammation) [20], this design constraint limits our ability to assess the Fr-ENS6Q’s diagnostic specificity in distinguishing ENS from other inflammatory nasal conditions. Although the Fr-ENS6Q demonstrated excellent discriminant validity between ENS patients and healthy controls (AUC = 0.989), patients with chronic rhinosinusitis or allergic rhinitis may present with similar symptoms, potentially yielding elevated Fr-ENS6Q scores. Future validation studies should include well-characterized disease control groups with documented absence of confounding comorbidities to establish disease-specific cutoff values and confirm the instrument’s clinical utility in differential diagnosis.

## 5. Conclusions

The Fr-ENS6Q is a valid and reliable patient-reported outcome questionnaire for assessing the severity of ENS symptoms in the French-speaking population. Further improvements to the ENS6Q could consider the inclusion of symptoms that are not present in the current ENS6Q.

## Figures and Tables

**Table 1:** Features of patients.

Features	N = 33
Age (mean, SD)	41.9 (12.7)
Gender (N, %)	
Females	14 (42.4)
Males	19 (57.6)
Diagnosis (N, %)	
Nasofibroscopy	16 (48.5)
Nasofibroscopy and CT scan	17 (51.5)
Cotton test	16 (48.5)
Self-diagnosis before confirmation	7 (21.2)
Etiologies	
Septoplasty and inferior turbinoplasty	12 (36.4)
Septoplasty, inferior turbinoplasty, and FESS	9 (27.3)
Turbinoplasty without septoplasty	8 (24.2)
Septorhinoplasty and inferior turbinoplasty	3 (9.1)
Frontal osteoma and middle turbinectomy	1 (3.0)
Comorbidities	
Laryngopharyngeal reflux disease	9 (27.3)
Allergy	8 (24.2)
Irritable bowel syndrome	8 (24.2)
Asthma	7 (21.2)
Migraine	7 (21.2)
Gastroesophageal reflux disease	5 (15.2)
Autoimmune disorders	4 (12.1)
Chronic obstructive pulmonary disease	3 (9.1)
Heart disease	3 (9.1)
Hypertension	3 (9.1)
Arthrosis	3 (9.1)
Osteoporosis	2 (6.1)
Thyroid disorder	1 (3.0)
Hypercholesterolemia	1 (3.0)
Severe sleep disorders (since the ENS onset)	21 (63.6)

Abbreviations: ENS = empty nose syndrome; N = number; SD = standard deviation.

**Table 2:** Prevalence and scores of patients and controls.

	ENS Patients		Asymptomatic Individuals		
Patient-Reported Outcome Questionnaires	Prevalence	Mean (SD)	Prevalence	Mean (SD)	*p*-Value
ENS6Q					
Dryness	33 (100)	4.4 ± 0.7	28 (46.0)	1.1 ± 1.2	0.001
Diminished nasal airflow	31 (93.9)	3.9 ± 1.4	27 (44.0)	1.3 ± 1.5	0.001
Suffocation	31 (93.9)	3.2 ± 1.7	10 (20.0)	0.4 ± 0.9	0.001
Nose feels too open	28 (84.8)	3.4 ± 1.8	4 (8.0)	0.2 ± 0.8	0.001
Nasal crusting	31 (93.9)	3.5 ± 1.4	29 (58.0)	1.2 ± 1.3	0.001
Nasal burning	28 (84.8)	3.2 ± 1.8	7 (14.0)	0.3 ± 0.9	0.001
ENS6Q total score	-	21.3 ± 6.1	-	4.5 ± 4.0	0.001
NOSE					
Nasal congestion or stuffiness	26 (78.8)	2.1 ± 1.4	29 (58.0)	1.2 ± 1.2	0.006
Nasal blockage or obstruction	30 (90.9)	2.7 ± 1.4	24 (48.0)	1.1 ± 1.4	0.001
Trouble breathing through my nose	32 (97.0)	3.5 ± 1.0	24 (48.0)	1.0 ± 1.2	0.001
Trouble sleeping	33 (100)	3.6 ± 0.9	27 (44.0)	0.7 ± 1.1	0.001
Unable to get enough air through my nose	29 (87.9)	3.1 ± 1.4	29 (58.0)	1.3 ± 1.3	0.001
NOSE total score	-	14.9 ± 4.3	-	5.2 ± 5.2	0.001
SNOT-22					
Blow nose	31 (93.9)	3.4 ± 1.4	38 (76.0)	1.8 ± 1.5	0.001
Sneezing	27 (81.8)	2.2 ± 1.5	33 (66.0)	1.7 ± 1.5	NS
Runny nose	23 (69.7)	1.8 ± 1.6	34 (68.0)	1.6 ± 1.4	NS
Cough	25 (75.6)	2.2 ± 1.7	26 (52.0)	0.8 ± 1.0	0.001
Postnasal discharge	30 (90.9)	3.5 ± 1.6	30 (60.0)	1.3 ± 1.3	0.001
Thick nasal discharge	30 (90.9)	2.9 ± 1.6	28 (46.0)	0.8 ± 1.2	0.001
Ear fullness	24 (72.7)	2.2 ± 1.6	24 (48.0)	1.0 ± 1.3	0.001
Dizziness	24 (72.7)	1.9 ± 1.6	22 (44.0)	0.9 ± 1.4	0.003
Ear pain	25 (75.6)	2.5 ± 1.8	18 (36.0)	0.9 ± 1.4	0.001
Facial pain	28 (84.8)	3.1 ± 1.7	15 (30.0)	0.6 ± 1.1	0.001
Difficulty falling asleep	32 (97.0)	3.7 ± 1.5	31 (62.0)	1.6 ± 1.6	0.001
Waking up at night	32 (97.0)	3.7 ± 1.6	40 (80.0)	2.2 ± 1.6	0.001
Lack of good night’s sleep	33 (100)	4.3 ± 1.0	41 (82.0)	2.6 ± 1.7	0.001
Walking up tred	33 (100)	4.5 ± 0.8	45 (90.0)	2.6 ± 1.6	0.001
Day fatigue	33 (100)	4.5 ± 0.8	44 (88.0)	2.5 ± 1.5	0.001
Reduced productivity	33 (100)	4.2 ± 1.0	39 (78.0)	2.2 ± 1.7	0.001
Reduced concentration	32 (97.0)	4.0 ± 1.3	29 (58.0)	2.3 ± 1.6	0.001
Irritability	33 (100)	4.0 ± 1.2	35 (70.)	1.9 ± 1.6	0.001
Sad	32 (97.0)	3.9 ± 1.4	33 (66.0)	1.8 ± 1.7	0.001
Embarrassed	33 (100)	4.4 ± 0.7	30 (60.0)	1.3 ± 1.4	0.001
Sense of taste/smell	27 (81.8)	2.8 ± 1.9	15 (30.0)	0.9 ± 1.6	0.001
Nose congestion	31 (93.9)	3.8 ± 1.5	34 (68.0)	1.6 ± 1.5	0.001
SNOT-22 total score	-	73.2 ± 18.0	-	34.6 ± 21.1	0.001

Abbreviations: ENS = empty nose syndrome; ENS6Q = Empty Nose Syndrome 6-Item Questionnaire; N = number; NOSE = Nasal Obstruction Symptom Evaluation; SD = standard deviation; SNOT-22 = Sinonasal Outcome Tool-22.

**Table 3:** Prevalence of depression and anxiety in patients.

Depression and Anxiety Outcomes	N (%)
Depression (PHQ-9) scores	
Minimal or none (0–4)	1 (3.0)
Mild (5–9)	3 (9.1)
Moderate (10–14)	1 (3.0)
Moderately severe (15–19)	13 (39.4)
Severe (20–27)	15 (45.5)
Patients requiring assessment (>14)	28 (84.9)
Anxiety (GAD-7) scores	
Minimal or none (0–4)	4 (12.1)
Mild (5–9)	5 (15.2)
Moderate (10–14)	8 (24.2)
Severe (15–21)	16 (48.5)
Patients requiring assessment (>9)	24 (72.7)

Abbreviations: GAD-7 = general anxiety disorder-7; PHQ-9 = Patient Health Questionnaire-9.

**Table 4:** Correlation analysis.

Outcome Measures	Spearman Coefficient (*p*-Value)			
ENS6Q	NOSE	SNOT-22	GAD-7	PHQ-9
Dryness	0.03 (NS)	0.19 (NS)	0.39 (0.025)	0.24 (NS)
Diminished nasal airflow	0.71 (0.001)	0.49 (0.004)	0.35 (0.043)	0.28 (NS)
Suffocation	0.51 (0.003)	0.65 (0.001)	0.58 (0.001)	0.59 (0.001)
Nose feels too open	0.33 (NS)	0.49 (0.005)	0.44 (0.012)	0.50 (0.003)
Nasal crusting	0.35 (0.048)	0.46 (0.008)	0.15 (NS)	0.02 (NS)
Nasal burning	0.36 (0.040)	0.52 (0.002)	0.22 (NS)	0.27 (NS)
Total ENS6Q	0.56 (0.001)	0.67 (0.001)	0.57 (0.001)	0.46 (0.001)

Abbreviations: ENS6Q = Empty Nose Syndrome 6-Item Questionnaire; GAD-7 = general anxiety disorder-7; N = number; NOSE = Nasal Obstruction Symptom Evaluation; NS = non-significant; PHQ-9 = Patient Health Questionnaire-9; SD = standard deviation; SNOT-22 = Sinonasal Outcome Tool-22.

## Data Availability

Data are available on request.
